# Automated digital TIL analysis (ADTA) adds prognostic value to standard assessment of depth and ulceration in primary melanoma

**DOI:** 10.1038/s41598-021-82305-1

**Published:** 2021-02-02

**Authors:** Michael R. Moore, Isabel D. Friesner, Emanuelle M. Rizk, Benjamin T. Fullerton, Manas Mondal, Megan H. Trager, Karen Mendelson, Ijeuru Chikeka, Tahsin Kurc, Rajarsi Gupta, Bethany R. Rohr, Eric J. Robinson, Balazs Acs, Rui Chang, Harriet Kluger, Bret Taback, Larisa J. Geskin, Basil Horst, Kevin Gardner, George Niedt, Julide T. Celebi, Robyn D. Gartrell-Corrado, Jane Messina, Tammie Ferringer, David L. Rimm, Joel Saltz, Jing Wang, Rami Vanguri, Yvonne M. Saenger

**Affiliations:** 1 Department of Medicine, Columbia University Irving Medical Center, P&S 9-428, 630 W 168th St., New York, NY 10032 USA; 2 Department of Anesthesiology, Perioperative Care and Pain Medicine, NYU School of Medicine, NYULMC, 435 E 30th St., SB-1008, New York, NY 10016 USA; 3 Vagelos College of Physicians and Surgeons, Columbia University, New York, NY USA; 4 Department of Dermatology, Pathology, and Oncological Sciences, Icahn School of Medicine at Mount Sinai, New York, NY USA; 5 Department of Dermatology, Columbia University Irving Medical Center, New York, NY USA; 6 Department of Biomedical Informatics, Stony Brook Medicine, Stony Brook, NY USA; 7 Department of Dermatology, University Hospitals Cleveland Medical Center/Case Western Reserve University School of Medicine, Cleveland, USA; 8 Oregon Health and Science University School of Medicine, Portland, OR USA; 9 Department of Pathology, Yale School of Medicine, New Haven, CT USA; 10 Department of Oncology and Pathology, Karolinska Institute, Stockholm, Sweden; 11 Department of Neurology, University of Arizona, Tucson, AZ USA; 12 Department of Medicine, Yale School of Medicine, New Haven, CT USA; 13 Department of Surgery, Columbia University Irving Medical Center, New York, NY USA; 14 Department of Pathology, University of British Columbia, Vancouver, Canada; 15 Department of Pathology, Columbia University Irving Medical Center, PH15-1582, 622 W 168th St., New York, NY 10032 USA; 16 Department of Pediatrics, Columbia University Irving Medical Center, New York, NY USA; 17 Department of Cutaneous Oncology, The Moffitt Cancer Center, Tampa, FL USA; 18 Department of Pathology, Geisinger Health System, Danville, PA USA; 19 Department of Neuroscience and Physiology, NYU School of Medicine, New York, NY USA

**Keywords:** Melanoma, Lymphocytes

## Abstract

Accurate prognostic biomarkers in early-stage melanoma are urgently needed to stratify patients for clinical trials of adjuvant therapy. We applied a previously developed open source deep learning algorithm to detect tumor-infiltrating lymphocytes (TILs) in hematoxylin and eosin (H&E) images of early-stage melanomas. We tested whether automated digital (TIL) analysis (ADTA) improved accuracy of prediction of disease specific survival (DSS) based on current pathology standards. ADTA was applied to a training cohort (n = 80) and a cutoff value was defined based on a Receiver Operating Curve. ADTA was then applied to a validation cohort (n = 145) and the previously determined cutoff value was used to stratify high and low risk patients, as demonstrated by Kaplan–Meier analysis (p ≤ 0.001). Multivariable Cox proportional hazards analysis was performed using ADTA, depth, and ulceration as co-variables and showed that ADTA contributed to DSS prediction (HR: 4.18, CI 1.51–11.58, p = 0.006). ADTA provides an effective and attainable assessment of TILs and should be further evaluated in larger studies for inclusion in staging algorithms.

## Introduction

There is an urgent need for prognostic biomarkers for high-risk early-stage melanoma. While it is established that immunotherapy is of benefit for advanced melanoma, defined as unresectable stage III and stage IV melanoma, the decision making for stage II and resectable stage III is more challenging for clinicians. In recent years, immunotherapies and targeted therapies have been approved in the adjuvant setting for Stage IIIA-D (lymph node positive) melanoma^1–6^. Clinical trials are underway for deep primary melanomas (Stage II). However, immunotherapy is associated with significant side effects and expense, with the yearly cost of immunotherapy for a single early-stage melanoma patient in the United States reaching over $100,000^7,8^. Further, the 5-year melanoma-specific survival (MSS) rates for patients with stage IIA-IIC range from 94 to 82%^9^. Thus, although some patients benefit from adjuvant therapy, treating all stage II-III patients would result in unnecessary expense and toxicity.

The current American Joint Committee on Cancer (AJCC) staging guidelines are used to clinically assess primary melanoma in order to predict the likelihood of recurrence and death from melanoma for the purpose of clinical decision making. Staging of the primary tumor (T stage) includes evaluation of Breslow thickness and ulceration, each of which is an independent predictor of MSS and recurrence-free survival (RFS)^10–12^. In this work we sought to test whether digital analysis of tumor infiltrating lymphocytes could add to current staging of primary melanoma tumors based on depth and ulceration.

Lymph node metastases are also commonly evaluated in staging (N stage) after T stage has been determined. However, lymph node dissection has not been shown to improve survival, confers some surgical risk, and in a minority of cases cannot be performed for anatomical reasons. Further, lymph node biopsy, while it provides prognostic information, is not always sufficient to independently guide therapy^13^. For example, Stage IIIA and IIIB melanoma patients (1–3 positive lymph nodes, 5-year MSS of 93% and 83%, respectively) live longer than stage IIC patients (node negative, 5-year MSS of 82%), complicating decisions to administer adjuvant therapy^9^. As such, it is crucial to develop readily clinically applicable biomarkers to improve risk assessment for early stage melanoma patients.

Many previous studies have sought to identify prognostic immune biomarkers for primary melanoma. Prognostic biomarkers that have been proposed in early-stage melanoma include Ki67 expression^14–16^, presence of driver mutations such as the BRAF mutation^17^, and gene expression profiles, one of which, based on the epithelial to mesenchymal transition, is commercially available^18,19^. Our research team has previously identified and validated a prognostic 53-gene signature (Melanoma Immune Profile, or MIP) that includes interferon-related genes, as well as a biomarker based on the ratio of cytotoxic T lymphocyte to macrophages within tumor stroma^18,20–22^. However, these biomarkers are all based on direct analysis of the tissue using immunohistochemical or genetic expression assays, a process which requires standardization across laboratory settings, and often mailing of specimens resulting in slow turn-around times. Further, because the initial biopsy that yields the diagnosis of melanoma is typically a small shave or punch biopsy specimen, tissue is often in limited supply and some specimens may become exhausted during the process of testing.

Biomarkers based on the analysis of Hematoxylin and Eosin (H&E) stained slides offer an alternative that facilitates the rapid estimation of prognostic risk and can be evaluated on electronically shared H&E images. In this study, we assess immune activity within the tumor using H&E images through quantitative evaluation of tumor-infiltrating lymphocytes (TILs). TILs, which are lymphocytes either in direct contact with tumor cells or that infiltrate the tumor nest, have been widely investigated as potential prognostic biomarkers in primary melanoma^10,11,23^. Pathologists currently use two methods to evaluate TIL density and distribution in the tumor microenvironment. A grade of 0, or absent, indicates an absence of TILs; a grade of 1 or 2, or non-brisk, indicates mild or moderate focal, mild multifocal, or mild diffuse TIL infiltrate; and a grade of 3, or brisk, indicates moderate diffuse or greater TIL infiltrate throughout the tumor region^10,23^. Several studies have found that the risk of recurrence is significantly greater for tumors with a TIL grade of 0 compared to those with a TIL grade of 3^23,24^. However, other studies have contested the validity of TILs as prognostic biomarkers because the qualitative evaluation of TILs is prone to intra- and inter-observer variability^25,26^. Despite the known role of the immune system in modulating tumor progression, the subjective nature of conventional TIL assessment and the variability in data obtained by pathologists at different academic centers have currently led to TILs not being included in standard AJCC staging methods^9^.

Digital pathology introduces a potentially more effective method to standardize TIL assessment and may minimize observer variability. Previous studies have sought to quantitatively automate analysis of TILs in cancer patients, including those with melanoma, but have not shown to improve accuracy of standard pathology evaluation^27,28^. A prior study employed a convolutional neural network (CNN), developed after training was performed on H&E whole slide images from the Cancer Genome Atlas (TCGA), which included thirteen tumor types including melanoma^28^. This deep learning computation method to identify lymphocytes in whole slide images is a major component of the National Cancer Institute-supported Quantitative Imaging in Pathology (QuIP) software suite. QuIP TIL CNN tiles images into patches and evaluates the probability of TILs in each tile, and, if a tile has a probability of TIL presence of at least 77.5%, it is considered a positive tile. A total score for each image is calculated based on the number of positive tiles over the total number of tiles. Each patient’s ADTA score is the median score of all of the patient’s images. The automated detection of lymphocytes generated quantitative assessments of TILs that highly correlated with molecular estimates of TILs in the TCGA samples in all of the different types of cancer^28^. The results generated by the QuIP TIL CNN were also validated by using ground truth labels generated by a panel of three pathologists. In this work, we employ this CNN to evaluate TILs in early-stage melanoma to predict disease specific survival (DSS) with ADTA.

## Results

### Automated digital TIL analysis (ADTA) correlates with standard pathology TIL assessment in primary melanoma tumors in training cohort

**Figure 1:**
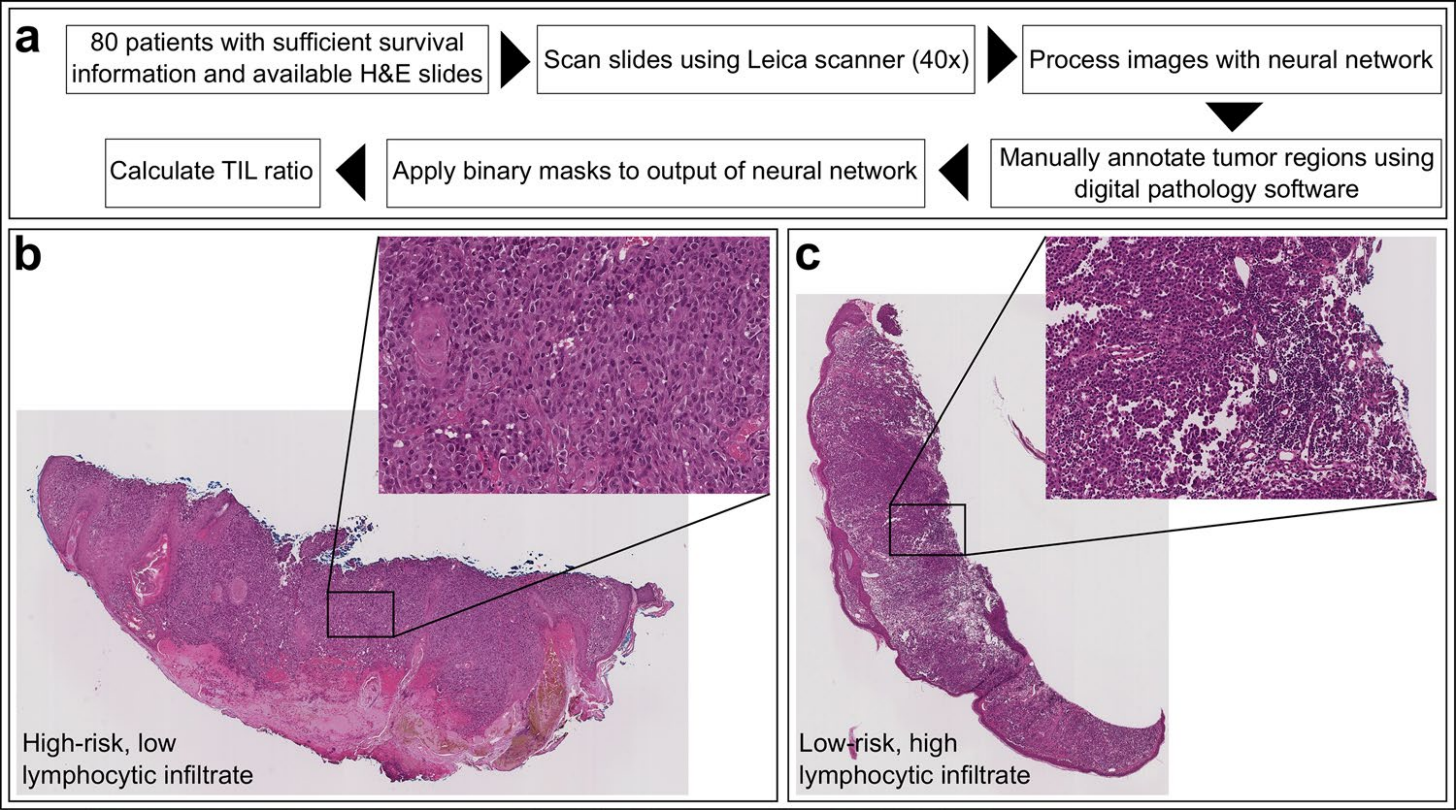
A detailed view of our approach. (**a**) Workflow for ADTA, based on Saltz et al. (QuPath v0.1.2: https://qupath.github.io/; QuIP: https://sbu-bmi.github.io/quip_distro/; TIL identification: https://github.com/SBU-BMI/quip_classification). Representative H&Es of (**b**) high-risk (low lymphocytic infiltrate) and (**c**) low-risk (high lymphocytic infiltrate) patients, as defined by the algorithm.

**Figure 2:**
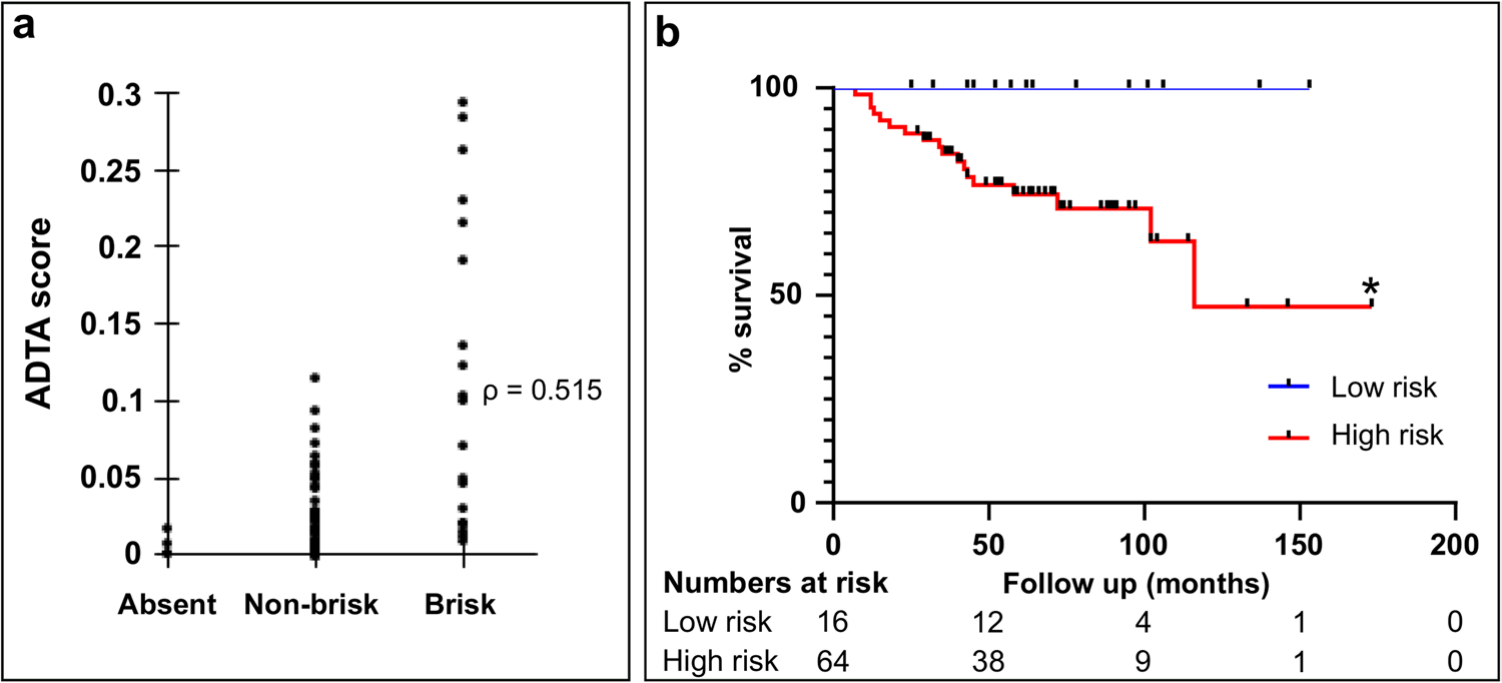
ADTA performance on training cohort. (**a**) ADTA score correlates with pathologist TIL grading defined as absent, non-brisk, or brisk (ρ = 0.515, p ≤ 0.001 using Spearman’s rank correlation coefficient). (**b**) KM curve for DSS created using ROC-defined cutoff (p = 0.0220 using log rank (Mantel Cox) test).

Images in the training cohort consisted of 80 subjects from Columbia University Irving Medical Center (CUIMC) diagnosed with primary melanoma tumors amenable to surgical resection between 2000 and 2014^29^. Demographics for the training population are shown in Table 1. Workflow for ADTA and representative H&E images are shown in Fig. 1. Features correlating with disease specific survival (DSS) by univariable Cox analysis in the training cohort included depth, TIL grade, and sentinel lymph node biopsy (SLNB) status (Depth: HR = 1.32, CI 0.78–2.25; p = 0.306; TIL grade: HR = 0.35, CI 0.00–0.95, p = 0.039; SLNB status: HR = 2.98, CI 1.04–8.55, p = 0.043, Supplemental Table S1) showing that this population generally conforms to trends observed in melanoma patients in the United States. TIL analysis by pathologists is complex and influenced by the growth phase and thickness of the melanoma^30^. Thus, in order to validate the ADTA method, TIL analysis was performed on these samples and correlated with TIL density as assessed by a pathologist using the criteria of brisk, non-brisk, and absent. Pathologists’ TIL grading for the training cohort correlated with ADTA (ρ = 0.515, p < 0.001, Fig. 2A).

**Table 1:** Patient characteristics of the training cohort.

(*n* = 80)	
**Clinical characteristics**	
**Sex, n (%)**	
Male	56 (70.0)
Female	24 (30.0)
**Age**	
Median, *n* (range)	67 (22–96)
**Location of tumor, n (%)**	
Trunk	45 (56.3)
Extremity	33 (41.2)
Unknown	2 (2.5)
**Pathologic characteristics**	
Depth (mm)	
Median, *n* (range)	2.0 (0.3–26.0)
**Ulceration, n (%)**	
Absent	43 (53.8)
Present	33 (41.2)
Unknown	4 (5.0)
**TILs**	
Absent	4 (5.0)
Non-brisk	50 (62.5)
Brisk	20 (25.0)
Unknown	6 (7.5)
**Microsatellite lesions, n (%)**	
Absent	77 (96.3)
Present	2 (2.5)
Unknown	1 (1.2)
**Staging characteristics**	
T-stage, *n* (%)	
T1a or T1b	19 (23.8)
T2a	9 (11.2)
T2b or T3a	32 (40.0)
T3b or T4a	16 (20.0)
T4b	4 (5.0)
**SLNB status, n (%)**	
Completed	44 (55.0)
Positive, *n* (% of completed)	11 (25.0)
Negative, *n* (% of completed)	33 (75.0)
Not completed	14 (17.5)
Unknown	22 (27.5)
**Stage, n (%)**	
I	19 (23.8)
II	48 (60.0)
III	13 (16.2)
**Outcome characteristics**	
**Patient follow-up (months)**	
Median, *n* (range)	58 (7–173)
**DMR, n (%)**	
Distant recurrence	21 (26.2)
No distant recurrence or local recurrence only	59 (73.8)
**OS, n (%)**	
Alive (at least 2 years)	55 (68.8)
Dead	25 (31.2)
**DSS, n (%)**	
Alive or NED at death	62 (77.5)
Median follow-up (months)	65.0
Dead with melanoma	18 (22.5)
Median follow-up (months)	34.5

*DMR* distant metastatic recurrence, *DSS* disease-specific survival, *NED* no evidence of disease, *OS* overall survival.

### Selection of cutoff for ADTA-based biomarker for prediction of disease specific survival (DSS)

TIL assessment by pathologists has generally shown correlation with clinical outcomes although accuracy has varied across institutions and the strength of these correlations has not been sufficient to include TILs in AJCC staging. In order to test whether ADTA correlated with patient outcomes and might provide additional information beyond standard pathology evaluation of the melanoma lesion, patients in the training set were divided into two groups based on whether or not they died of melanoma. In order to develop a clear metric for clinical application, a Receiver Operating Characteristic curve (ROC) was used to define a cutoff most accurately distinguishing patients who died of melanoma from those who did not. When the cutoff, 0.065, was applied to the training cohort, Kaplan Meier (KM) analysis showed that the binary ADTA score correlated with DSS (p = 0.0220, Fig. 2B). This cutoff was then defined as the basis for determining high and low-risk groups in the validation set.

### ADTA biomarker correlates with DSS in validation cohort

**Figure 3:**
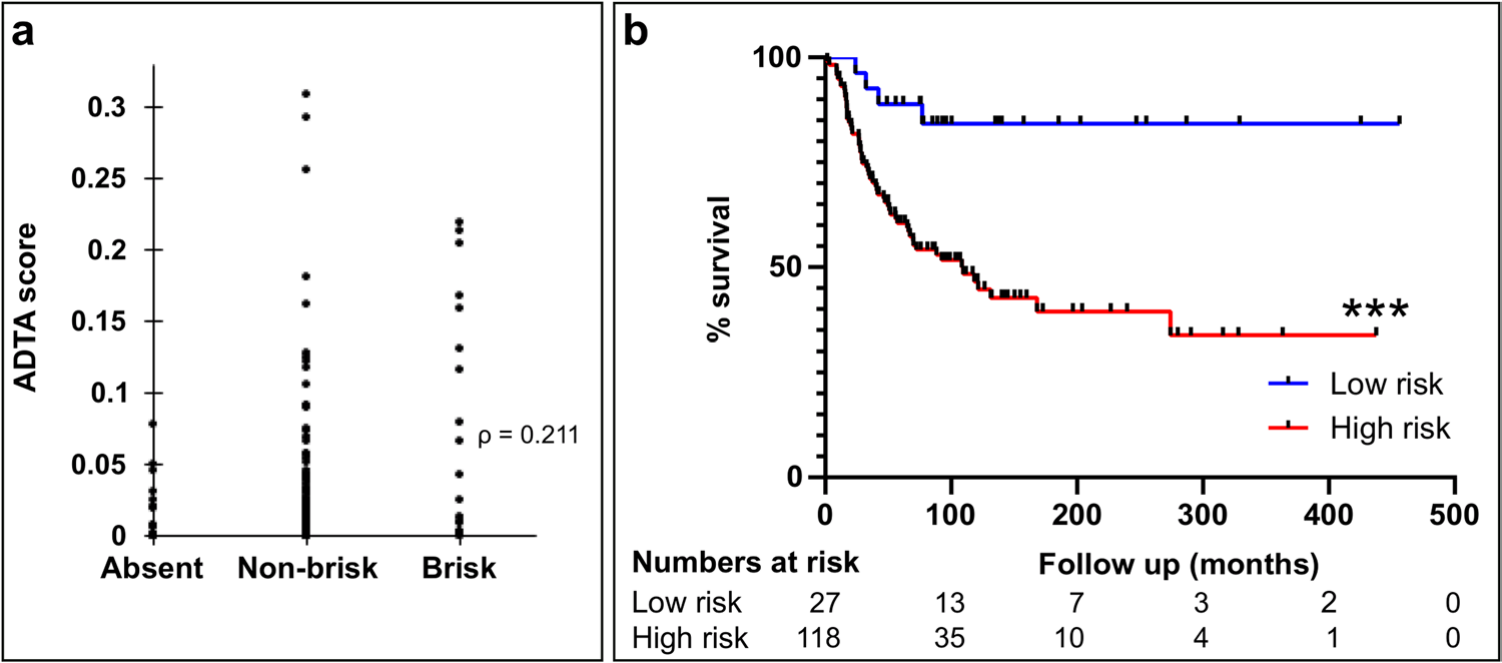
ADTA performance on validation cohort. (**a**) Correlation between ADTA score and pathologist TIL grading defined as absent, non-brisk, or brisk (ρ = 0.211, p = 0.011 using Spearman’s rank correlation coefficient). (**b**) KM curve for DSS created using pre-defined cutoff (p < 0.001 using log rank (Mantel Cox) test).

The validation cohort consisted of patients from Yale School of Medicine (YSM) and Geisinger Health Systems (GHS) with demographics shown in Table 2 (N = 145). Demographics for patients from each institution are shown separately in Supplemental Table S2. (YSM, N = 100, GHS, N = 45). ADTA correlated weakly with pathologist TIL grading in the validation set (ρ = 0.211, p = 0.011, Fig. 3A). ADTA correlated more strongly with pathologist TIL grading for each population separately (ρ = 0.345, p < 0.001 for YSM and ρ = 0.354, p = 0.019 for GHS, Supplemental Fig. S1), demonstrating difficulties in combining TIL scoring from multiple pathologists. Additionally, a significant difference was found among ADTA scores when patient ADTA scores were stratified by the patients’ corresponding TIL grade (p < 0.0001, Supplemental Fig. S2). ADTA score distributions further stratified by TIL grade within cohorts and institutions are shown in Supplemental Fig. S2. KM analysis showed that the binary ADTA score effectively correlated with DSS (p < 0.001, Fig. 3B). As shown in Fig. 3B, the number of patients at risk at 0 months of follow up was 27 and 118 for the low-risk and high-risk groups, respectively. The number of patients at risk at 100 months of follow up was 13 and 35 for the low-risk and high-risk groups, respectively. KM analysis separating the validation cohort by institution shows that accuracy of DSS prediction was significant in YSM and GHS populations (p = 0.0139 and p = 0.0141, Supplemental Fig. S3). The data shows that the ADTA biomarker correlated with DSS in the combined validation cohort and that results were consistent across both populations.

**Table 2:** Patient characteristics of the validation cohort.

(*n* = 145)	
**Clinical characteristics**	
Sex, *n* (%)	
Male	72 (49.7)
Female	73 (50.3)
**Age**	
Median, *n* (range)	62 (20–90)
**Location of tumor, n (%)**	
Trunk	29 (20.0)
Extremity	16 (11.0)
Unknown	100 (69.0)
**Pathologic characteristics**	
Depth (mm)	
Median, *n* (range)	2.75 (0.15–13.00)
**Ulceration, n (%)**	
Absent	81 (55.9)
Present	64 (44.1)
Unknown	0 (0.0)
**TILs**	
Absent	17 (11.7)
Non-brisk	108 (74.5)
Brisk	19 (13.1)
Unknown	1 (0.7)
**Microsatellite lesions, n (%)**	
Absent	114 (78.6)
Present	31 (21.4)
Unknown	0 (0.0)
**Staging characteristics**	
T-stage, *n* (%)	
T1a or T1b	23 (15.9)
T2a	14 (9.7)
T2b or T3a	44 (30.3)
T3b or T4a	40 (27.6)
T4b	24 (16.5)
**SLNB status, n (%)**	
Completed	41 (28.3)
Positive, *n* (% of completed)	19 (46.3)
Negative, *n* (% of completed)	22 (53.7)
Not completed	4 (2.8)
Unknown	100 (68.9)
**Stage, n (%)**	
I	31 (21.4)
II	66 (45.5)
III	48 (33.1)
**Outcome characteristics**	
Patient follow-up (months)	
Median, *n* (range)	72.5 (1.4–456.2)
**DMR, n (%)**	
Distant recurrence	69 (47.6)
No distant recurrence or local recurrence only	76 (52.4)
**OS, n (%)**	
Alive (at least 2 years)	97 (66.9)
Dead	48 (33.1)
**DSS, n (%)**	
Alive or NED at death	82 (56.6)
Median follow-up (months)	99.8
Dead with melanoma	63 (43.4)
Median follow-up (months)	33.0
Unknown	0 (0.0)

*DMR* distant metastatic recurrence, *DSS* disease-specific survival, *NED* no evidence of disease, *OS* overall survival.

### ADTA risk status enhances standard pathology assessment methods, improving accuracy of survival prediction based on depth and ulceration

**Figure 4:**
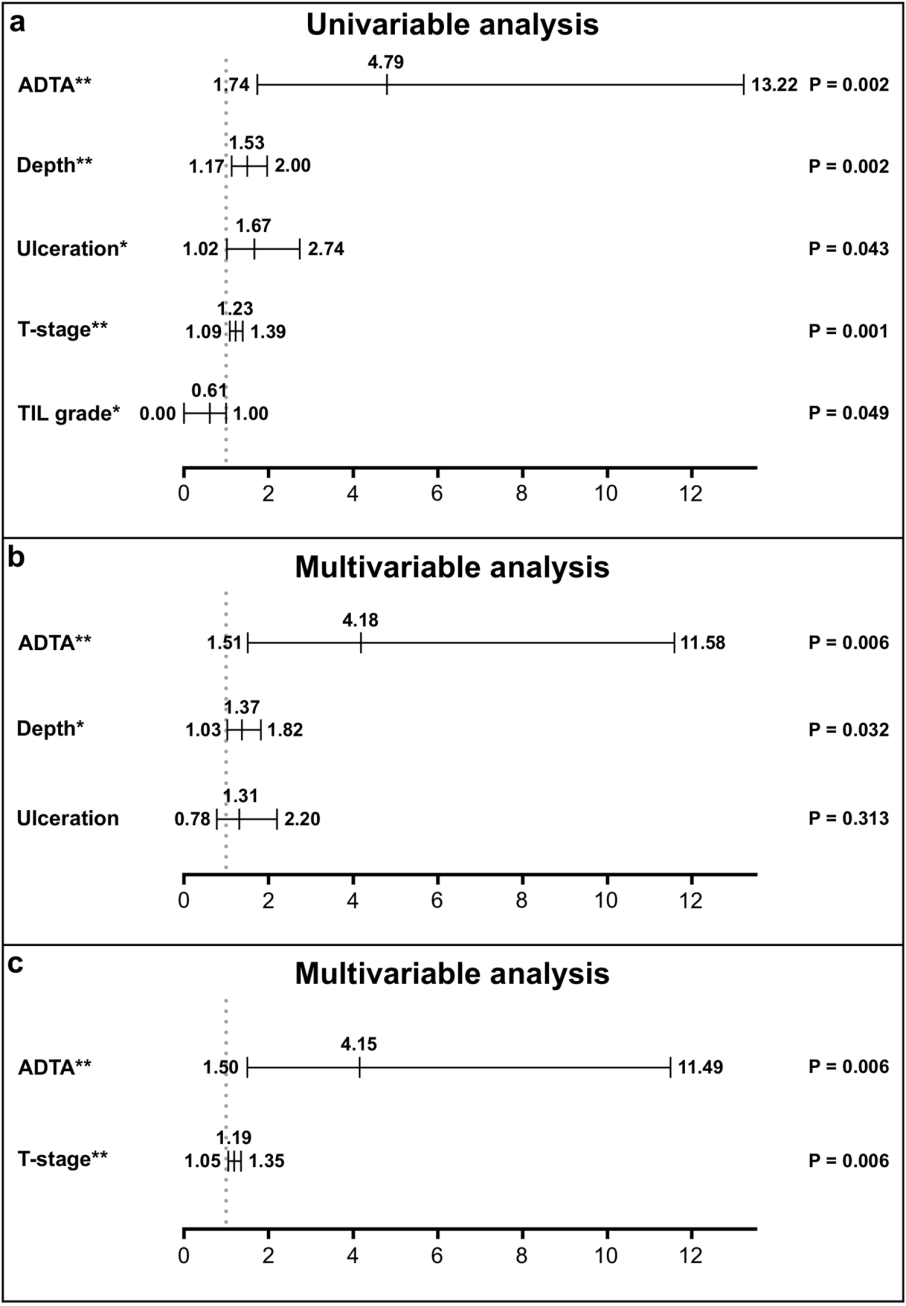
Cox regression analysis of validation cohort. (**a**) Univariable Cox regression analysis of disease-specific survival on validation cohort including ADTA, depth, ulceration, T-stage, and TIL grade. (**b**) Multivariable Cox regression analysis of disease-specific survival on validation cohort including ADTA, depth, and ulceration. (**c**) Multivariable Cox regression analysis of disease-specific survival on validation cohort including ADTA and T-stage.

Within the validation set, depth, ulceration, T stage, and TIL grade correlated with DSS by univariable analysis (depth: HR = 1.53, CI: 1.17–2.00, p = 0.002; ulceration: HR = 1.67, CI 1.02–2.74, p = 0.043; T stage: HR = 1.23, CI 1.09–1.39, p = 0.001; TIL grade: HR = 0.61, CI 0.00–1.00, p = 0.049, Fig. 4A). ADTA correlated with DSS by univariable analysis (HR = 4.79, CI 1.74–13.22, p = 0.002, Fig. 4A). Univariable analysis separating the validation cohort by institution is shown in Supplemental Table S3. A multivariable Cox proportional hazards model performed using depth and ulceration as co-variables showed that ADTA contributed significantly to DSS prediction (HR = 4.18, CI 1.51–11.58, p = 0.006, Fig. 4B). In contrast, a multivariable Cox proportional hazards model including conventional pathologist TIL grading, depth, and ulceration found that only depth significantly added to the model (HR = 1.40, CI 1.03–1.89, p = 0.031, Supplemental Table S4). Notably, when T stage, which uses depth and ulceration as inputs, was used as a covariable, ADTA significantly improved accuracy of the overall model (HR = 4.15, CI 1.50–11.49, p = 0.006, Fig. 4C). These results show that ADTA adds value to pathology evaluation of the primary tumor. Most patients with deep primary melanomas have a surgical biopsy of the sentinel lymph node procedure followed by, in many cases, a completion dissection if the sentinel lymph node is positive. Because staging protocols evolved over time, a significant proportion of patients in our validation cohort did not have sentinel lymph node procedures and were staged clinically. However, in order to estimate whether ADTA added to final staging, based on available information in our medical records, we ran a multivariable cox analysis with stage as a co-variable. Any patient with a known positive lymph node or documented satellite metastasis was scored as a stage III and patients without these findings were scored as stage I or II based on depth. ADTA significantly improved accuracy of the overall model (HR = 4.61, CI 1.67–12.71, p = 0.003, Supplemental Table S5). This data shows that ADTA enhanced the predictive value of standard pathology features of depth and ulceration in the validation set, outperforming conventional dermato-pathologist assessment using depth and ulceration.

## Discussion

Digital pathology is gaining prominence in modern clinical practice and will likely become crucial to diagnosis in the near future. In this work, we show that digital pathology images can be analyzed to provide TIL estimates that improve standard pathology assessments and have potential to contribute meaningfully to clinical care. This is the first report, to our knowledge, that digital analysis of TILs not only correlates with survival, but adds to standard pathology predictors. ADTA contributed significantly to prognostic accuracy in the context of clinical predictors using multivariable Cox analysis (p = 0.006, Fig. 4B) whereas standard qualitative TIL analysis by a dermato-pathologist did not (p = 0.323, Supplemental Table S4). This method is distinct from a previous AI based prognostic developed by our group using a convolutional neural network^29^ in that it focuses exclusively on TILs and represents a new application of a previously developed open source software and thus can be directly applied to clinical samples by pathologists^28^.

ADTA requires pathologist supervision. This work was done with supervision of a pathologist blinded to clinical outcomes (G.N.) as depending on tumor cell morphology, the distinction between tumor and surrounding tissue is difficult to determine for untrained personnel. The need for pathologist supervision poses a limitation to the rapidity of ADTA and introduces some user variability, as the area determined to be the tumor region could differ depending on the pathologist. This could lead to slightly varying cutoff values given that the cutoff is determined based on the scores assigned to each patient, which rely on the specified tumor region. Going forward, developing a method to automate the identification of the tumor region would eliminate the need for pathologist supervision, reduce user variability, and provide more uniformity across cohorts and users. Furthermore, the ability for a single script to run the QuIP TIL CNN and subsequently calculate the median value over all images for a given patient would reduce manual processes. Interestingly, while ADTA correlated with pathologist-assessed TILs, it was more closely associated with outcome. Notably, ADTA is not identical to pathology assessment of TILs as it includes evaluation of the entire tumor area rather than focusing on the vertical growth phase^31^. ADTA may correlate better than pathology assessment across institutions because, while ADTA correlated with pathologists’ assessment, individual pathologists may have slightly different standards of classification leading to difficulties combining datasets as demonstrated by the fact that correlation weakened when we combined our two validation sets. Further, ADTA allows for a precise cutoff to be defined which may enhance detection of the threshold of TILs required to provide meaningful evidence of anti-tumor immunity, rather than relying on qualitative differences between brisk and non-brisk. Lastly, ADTA has the advantage of allowing for standardization and quantification across institutions. Data from the three populations included in this study suggest that the algorithm has potential to be readily applied to H&E images across institutions, an important consideration for application to clinical care.

One limitation of the dataset is that the groups are unbalanced; only a minority of patients (19%) in the validation set fell into the good prognosis group. This group did quite well compared to the high-risk group. In the low-risk group, 15% of patients died of melanoma, of whom 0% died in less than two years. In the high-risk group, 50% died of melanoma, of whom 36% (14% of total) died within two years. This data is consistent with prior results suggesting that high TIL infiltration is protective for the minority of patients who fall into the good prognosis group and may reflect the biologic implications of high levels of infiltrating lymphocytes. Notably, for standard TIL analysis performed at a single institution, similar data has been reported with a minority of patients having higher TILs indicative of favorable outcome^23^.

A second limitation is that, due to changes in practice over time and local preference, many patients did not have sentinel lymph node procedures performed. Thus, while we conclude that ADTA improves pathology assessment of the primary tumor, we cannot determine whether ADTA adds value to complete surgical staging. Certainly, however, from the clinician and patient’s standpoint, it does appear to be desirable to obtain as much clinically relevant data as possible from the original biopsy in addition to proceeding with surgical resection of lymph nodes, a procedure that carries some, if minimal, risk particularly for elderly patients. Further, in the real-world setting, patients are increasingly opting against completion lymph node dissection (CLND) and, in some cases, SLNB^32^. Additional studies are required to address the value of ADTA in the context of complete lymph node staging. Finally, it must be noted that additional information regarding the phenotype of TILs can be obtained using staining methods including simple immunohistochemistry and quantitative immune-fluorescence. While direct analysis of H&E can only quantify gross lymphocyte infiltration, it is readily applicable to the diagnostic slides from any biopsy and thus simpler to apply clinically than more complex staining protocols. Further, there is currently no well validated staining based prognostic biomarker in early stage melanoma, although several are under development^20,33^.

In summary, the above data strongly suggests that ADTA may be superior to conventional qualitative TIL assessment particularly over larger multi-institution cohorts and be sufficiently useful to include in standard pathology evaluation of melanomas and possibly in AJCC staging. As digital pathology becomes more broadly utilized, TIL algorithms, such as the open source QuIP TIL CNN software, may be further developed into apps and included in the digital process as part of standard staging. Such apps would provide additional prognostic information at minimal cost. Further assessment on larger databases is warranted as it has the potential to provide patients with more accurate assessment of their risk of dying of melanoma, and would be relatively straightforward to perform.

## Methods

### Clinical information and patients

This project was approved by CUIMC’s Institutional Review Board (IRB) and was determined not to necessitate written consent from subjects as the study is retrospective and of low risk; therefore, informed consent was waived by the ethics committee (CUIMC’s IRB). This experiment was conducted in agreement with the ethical guidelines outlined by the Declaration of Helsinki. Subjects were obtained from previously generated databases for a study concerning the development of a deep learning algorithm to predict melanoma recurrence^29^. Subjects were selected based on the criteria that there was at least one available H&E slide and at least 24 months of clinical follow up information, unless the subject died of melanoma. All patients included had available distant metastatic recurrence (DMR) information. Complete patient demographics for the training cohort are found in Table 1. The validation cohort consisted of patients from two institutions: Yale School of Medicine (YSM, N = 100, Supplemental Table S2) and Geisinger Health System (GHS, N = 45, Supplemental Table S2). The complete patient demographics for the patients in this validation cohort are found in Table 2. Patients were characterized based on whether they died of melanoma over the follow up period^29^.

### Imaging

Primary melanoma biopsies were collected and lymph node biopsies were excluded. All slides used in this project were reviewed by a dermato-pathologist from each institution to confirm the presence of melanoma and assess TIL grade. Slides were scanned using LEICA SCN 400 system with a high throughput 384 slide auto-encoder (SL801) to generate .scn images at 40x (CUIMC, GHS) or using Aperio ScanScope XT platform (Leica Biosystems) to generate. svs images at 20 × (YSM). Many patients had multiple whole slide images for one tumor, as separate images were generated for distinct areas of melanoma tissue. This is frequently the case in primary melanomas due to tissue sectioning methods. Ten patients were excluded from the training cohort due to the presence of excessive melanin, which obscured the image, and one patient was excluded because the tissue was torn. One patient was excluded from the validation cohort because the size of the image was incompatible with QuPath, the program used to create the binary masks.

### Analysis pipeline

The QuIP TIL CNN (https://github.com/SBU-BMI/quip_classification) was employed using Python 3.5 and TensorFlow 1.8 to analyze both the training and validation cohorts and was run on Ubuntu 16.04 (CPU: Intel Xeon W-2195 @ 2.30 GHz; GPU: NVIDIA GP102GL [Quadro P6000]). The algorithm tiled each image into 100 × 100 pixel patches and evaluated the probability that lymphocytes exist in each patch. For each image the algorithm generated a file with the x and y coordinates of the upper left vertex and the probability of lymphocytic infiltration associated with each patch. Each H&E image in the analysis was manually annotated with a loop drawn specifically around tumor areas in QuPath 0.1.2 (https://qupath.github.io/), an open source digital pathology program that allows visualization of H&E images^34^. Image annotation analysis was performed by a technician under the supervision of a dermato-pathologist (G.N.). Binary masks were then generated using the annotations in QuPath and applied to the output files of the deep learning algorithm to consider only probabilities of patches inside the tumor region. Patches with a probability of lymphocytic infiltration above 77.5%, an empirically determined threshold by the creators of the algorithm, were considered “positive” for lymphocytes. The “ADTA Score” (# of TIL positive patches in the tumor/# of total patches in the tumor) was then calculated for each image. Detailed method was previously published^28^. Each patient was assigned a score based on the median value of the TIL ratios for all images assigned to the patient.

### Statistics

Statistical analysis was performed using XLSTAT Version 2019.1.3 on Excel Version 15.0.5127 and GraphPad Prism Version 8.0.1. Statistical significance was defined at p ≤ 0.05. Receiver Operating Curves (ROC) and univariable and multivariable Cox proportional hazards models were created using the “Survival Analysis” tool in XLSTAT. Kaplan Meier (KM) curves were created on GraphPad prism and p values were determined using log-rank (Mantel-Cox) test. Spearman correlation coefficients were used to evaluate correlation between pathologists’ TIL grading and the ADTA score.

## Supplementary Information

Supplementary Information

## Data Availability

All datasets analyzed during the current study are available from the corresponding author on reasonable request.
