# Supplementary material for: Automated digital TIL analysis (ADTA) adds prognostic value to standard assessment of depth and ulceration in primary melanoma
Source: Sci Rep. 2021 Feb 2;11:2809. doi: 10.1038/s41598-021-82305-1 (PMC7854647; doi:10.1038/s41598-021-82305-1)
Supplement: Supplementary file 1 — Supplementary Information [file 41598_2021_82305_MOESM1_ESM.pdf]

Title: Automated digital TIL analysis (ADTA) adds prognostic value to standard assessment of depth and ulceration in primary melanoma

Authors: Michael R. Moore*, Isabel D. Friesner*, Emanuelle M. Rizk*, Benjamin T. Fullerton, Manas Mondal, Megan H. Trager, Karen Mendelson, Ijeuru Chikeka, Tahsin Kurc, Rajarsi Gupta, Bethany R. Rohr, Eric J. Robinson, Balazs Acs, Rui Chang, Harriet Kluger, Bret Taback, Larisa J. Geskin, Basil Horst, Kevin Gardner, George Niedt, Julide T. Celebi, Robyn D. Gartrell-Corrado, Jane Messina, Tammie Ferringer, David L. Rimm, Joel Saltz, Jing Wang**, Rami Vanguri**, Yvonne M. Saenger**

*These authors contributed equally to this work
**These authors contributed equally to this work

Supplemental Table 1. Univariable Cox regression analysis of disease-specific survival on Training cohort, including pathology TIL grade, depth, ulceration, and sentinel lymph node biopsy (SLNB) status.

Univariable cox analysis using training cohort.

	Hazard ratio	95% CI	*P*
**Pathology TIL grade***	0.35	0.00-0.95	0.039
**Depth**	1.32	0.78-2.25	0.306
**Ulceration**	2.47	0.91-6.73	0.076
**SLNB***	2.98	1.04-8.55	0.043

Supplemental Table 2. Validation demographics, separated by institution**.**

	Yale	Geisinger
**Clinical characteristics**	**(n = 100)**	**(n = 45)**
Sex, *n* (%)		
Male	48 (48.0)	24 (53.3)
Female	52 (52.0)	21 (46.7)
Age		
Median, *n* (range)	61 (25-84)	65 (20-90)
Location of tumor, *n* (%)		
Trunk	N/A	29 (64.4)
Extremity	N/A	16 (35.6)
**Pathologic characteristics**		
Depth (mm)		
Median, *n* (range)	2.3 (0.2-8.3)	3.5 (0.7-13.0)
Ulceration, *n* (%)		
Absent	61 (61.0)	25 (55.6)
Present	39 (39.0)	20 (44.4)
Unknown	0 (0.0)	0 (0.0)
TILs		
Absent	6 (6.0)	11 (24.5)
Non-brisk	76 (76.0)	32 (71.1)
Brisk	18 (18.0)	1 (2.2)
Unknown	0 (0.0)	1 (2.2)
Microsatellite lesions, *n* (%)		
Absent	75 (75.0)	39 (86.7)
Present	25 (25.0)	6 (13.3)
Unknown	0 (0.0)	0 (0.0)
**Staging Characteristics**		
T-stage, *n* (%)		
T1a or T1b	22 (22.0)	1 (2.2)
T2a	14 (14.0)	0 (0.0)
T2b or T3a	28 (28.0)	16 (35.6)
T3b or T4a	22 (22.0)	18 (40.0)
T4b	14 (14.0)	10 (22.2)
Unknown	0 (0.0)	0 (0.0)
SLNB status, *n* (%)		
Completed	0 (0.0)	40 (88.9)
Positive, *n* (% of completed)	0 (0.0)	20 (50.0)
Negative, *n* (% of completed)	0 (0.0)	20 (50.0)
Not completed	0 (0.0)	5 (11.1)
Unknown	100 (100.0)	0 (0.0)
Stage, *n*(%)		
I	30 (30.0)	1 (2.2)
II	45 (45.0)	21 (46.7)
III	25 (25.0)	23 (51.1)
**Outcome characteristics**		
Patient follow-up (months)		
Median, *n* (range)	68.7 (1.4-456.2)	76.0 (9-142)
DMR, *n* (%)		
Distant recurrence	44 (44.0)	25 (55.6)
No distant recurrence or local recurrence only	56 (56.0)	20 (44.4)
OS, *n* (%)		
Alive (at least 2 years)	26 (26.0)	22 (48.9)
Dead	74 (74.0)	23 (51.1)
DSS, *n* (%)		
Alive or NED at death	56 (56.0)	26 (57.8)
Median follow-up (months)	114.4	94.0
Dead with melanoma	44 (44.0)	19 (42.2)
Median follow-up (months)	39.0	28.0
Unknown	0 (0.0)	0 (0.0)

Abbreviations: DMR, distant metastatic recurrence; DSS, disease-specific survival; NED, no evidence of disease; OS, overall survival

Supplemental Figure 1. ADTA correlated with pathologist TIL grading. Correlation between ADTA score and pathologist TIL grading defined as absent, non-brisk, or brisk for (a) Yale cohort (ρ=0.345, p<0.001 using Spearman's rank correlation coefficient) and (b) Geisinger cohort (ρ=0.354, p=0.019 using Spearman's rank correlation coefficient).

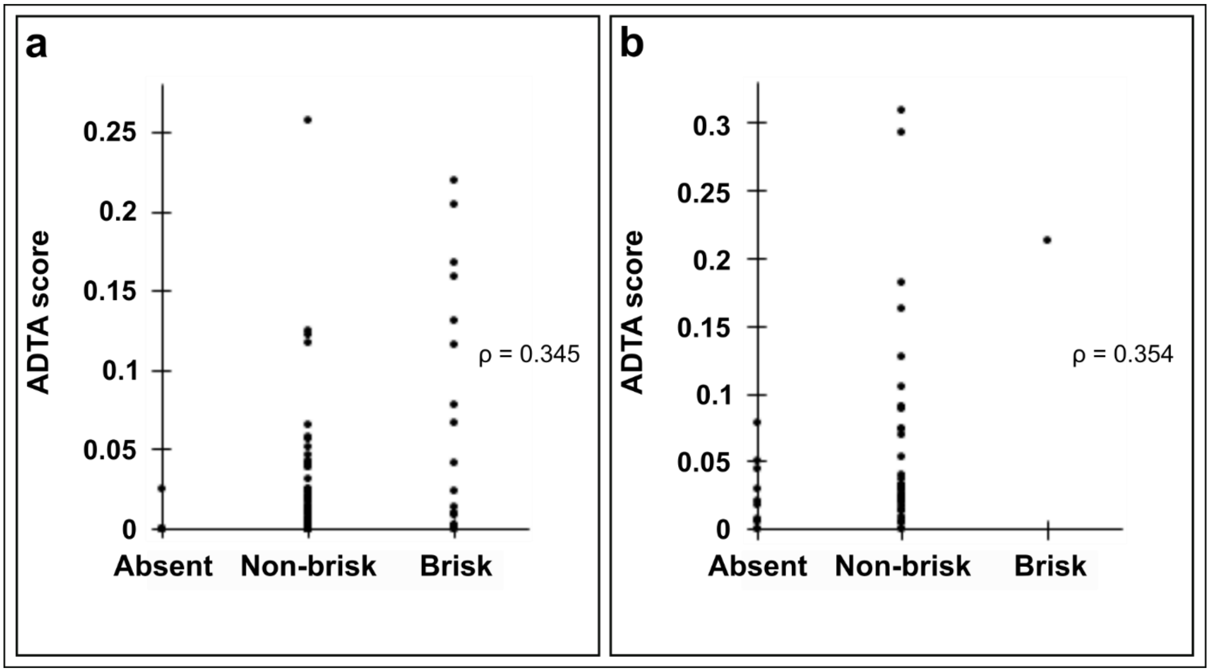

Supplementary Figure 2. ADTA score distributed by pathologist TIL grade. (a) Analysis of variance between ADTA scores of all patients stratified by their corresponding patient TIL grade (Kruskal-Wallis, p<0.0001; Dunn multiple comparison p values of 0.1850 for Absent vs. Non-brisk, <0.0001 for Absent vs. Brisk, and 0.0002 for Non-brisk vs. Brisk). (b) Distribution of ADTA scores by TIL grade within the training (CUIMC) and validation (GHS and YMS) cohorts. (c) Distribution of ADTA scores by TIL grade within each institution.

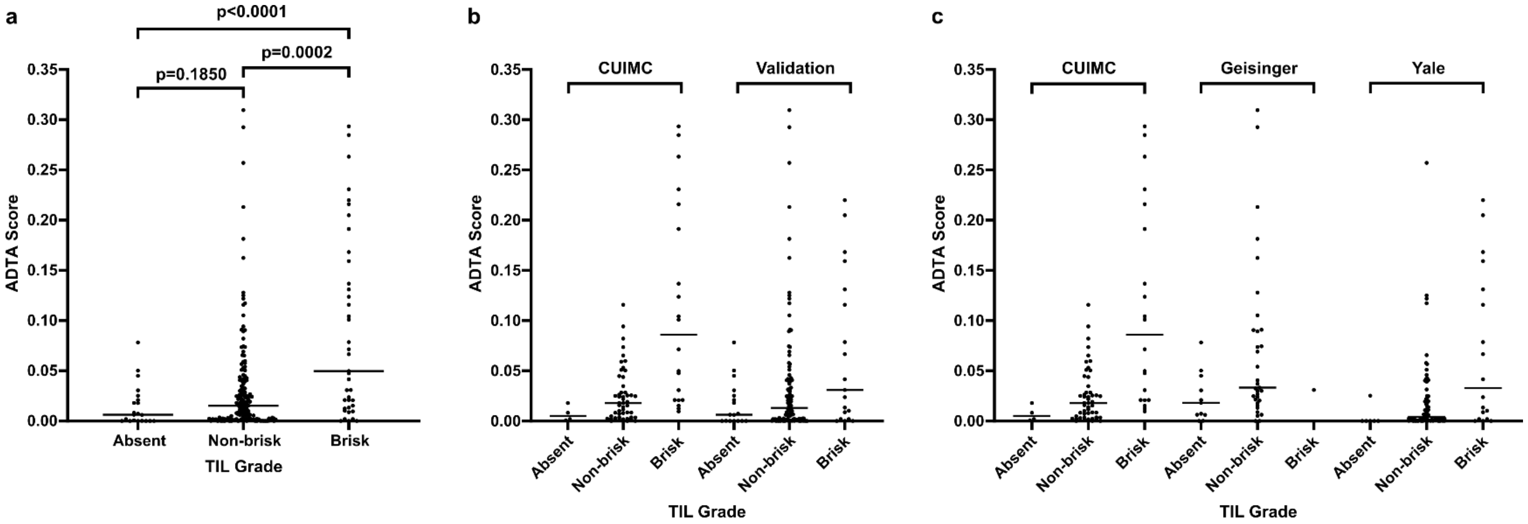

Supplemental Figure 3. ADTA performance on Yale and Geisinger populations, individually. KM curves for disease-specific survival on (a) Yale population (p=0.0139 using log rank (Mantel-Cox) test) and (b) Geisinger population (p=0.0141 using log rank (Mantel-Cox) test).

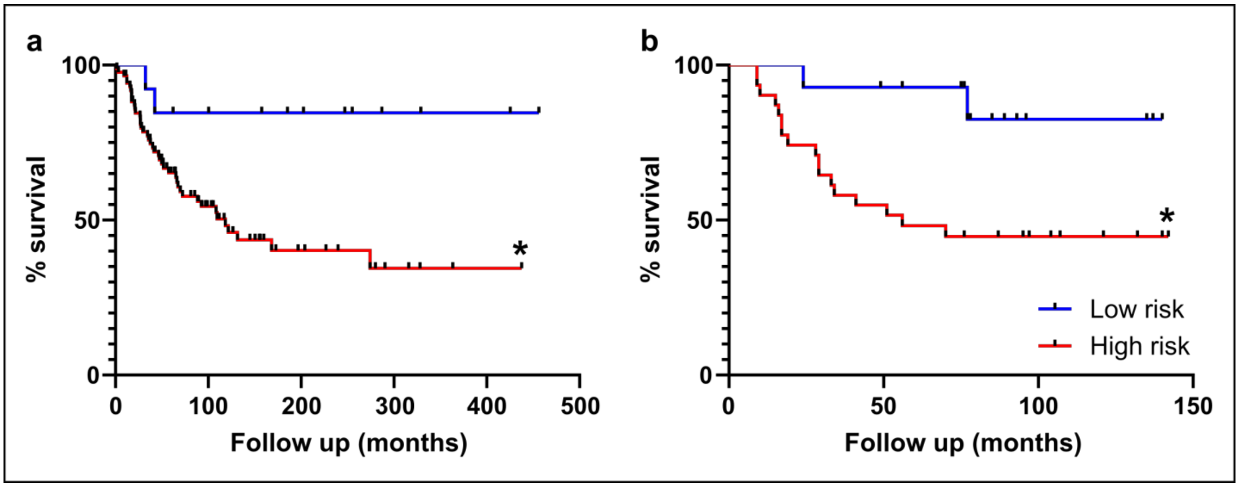

Supplemental Table 3. Univariable Cox regression analysis of disease-specific survival on Yale and Geisinger cohorts, including ADTA, depth, ulceration, T-stage, pathology TIL grade, and SLNB status.

Univariable cox analysis using validation cohort.

	Yale		Geisinger	
	**Hazard ratio (95% CI)**	***P***	**Hazard ratio (95% CI)**	***P***
**ADTA**	5.01 (1.21-20.80)	0.027	5.17 (1.19-22.41)	0.028
**Depth**	1.59 (1.17-2.16)	0.003	1.54 (0.76-3.11)	0.231
**Ulceration**	1.34 (0.74-2.44)	0.333	2.86 (1.03-7.96)	0.044
**T-Stage**	1.22 (1.07-1.40)	0.004	1.36 (0.97-1.90)	0.071
**Pathology TIL grade**	0.50 (0.00-0.95)	0.035	0.78 (0.31-1.98)	0.598
**SLNB**	N/A	N/A	1.51 (0.57-3.97)	0.406

Supplemental Table 4. Multivariable Cox regression analysis of disease-specific survival on validation cohort, including pathology TIL grade, depth, and ulceration.

Multivariable cox analysis using validation cohort.

	**Hazard ratio**	**95% CI**	***P***
**Pathology TIL grade**	0.79	0.46-1.38	0.414
**Depth***	1.40	1.03-1.89	0.031
**Ulceration**	1.29	0.77-2.18	0.335

Supplemental Table 5. Multivariable Cox regression analysis of disease-specific survival on validation cohort, including ADTA and stage.

Multivariable cox analysis using validation cohort.

	**Hazard ratio**	**95% CI**	***P***
**ADTA****	4.61	1.67-12.71	0.003
**Stage****	1.77	1.24-2.53	0.002
